# Insight of the role of mitochondrial iron homeostasis in insulin resistance

**DOI:** 10.7717/peerj.21873

**Published:** 2026-11-02

**Authors:** Zhanchen Dong, Yan Jiang

**Affiliations:** 1 Evidence-Based Nursing Research Laboratory, West China Hospital, Sichuan University/West China School of Nursing, Sichuan University, Chengdu, Sichuan, China; 2 Department of Nursing, West China Hospital, Sichuan University/West China School of Nursing, Sichuan University, Chengdu, Sichuan, China

**Keywords:** Mitochondrial iron, Mitoferrin, Redox system, Mitochondrial dysfunction, Insulin resistance

## Abstract

Insulin resistance (IR) is characterized by impaired insulin responsiveness in metabolic tissues, leading to defective glucose disposal, dysregulated lipid metabolism, and compensatory hyperinsulinemia. Mitochondrial iron homeostasis is crucial for cellular metabolism, oxidative phosphorylation (OXPHOS), and redox balance. Dysregulation of mitochondrial iron contributes to IR by impairing electron transport chain (ETC) function, increasing reactive oxygen species (ROS), and disrupting insulin signaling pathways. Excess mitochondrial iron leads to oxidative stress through the Fenton reaction, promoting lipid peroxidation, mitochondrial dysfunction, and inflammation, all of which exacerbate metabolic disorders. Additionally, mitochondrial iron overload affects iron-sulfur (Fe-S) cluster biosynthesis, mitochondrial dynamics, and energy metabolism, further impairing glucose homeostasis. Emerging evidence suggests that mitochondrial iron dysregulation plays a pivotal role in the pathogenesis of type 2 diabetes mellitus (T2DM) and metabolic syndrome. Future research should focus on the molecular mechanisms governing mitochondrial iron transport and utilization, as well as its interaction with key metabolic pathways, to better understand its role in disease progression and potential therapeutic implications.

## Introduction

Insulin resistance (IR) is a pathological metabolic state characterized by reduced responsiveness of insulin-sensitive tissues, including skeletal muscle, liver, and adipose tissue, to insulin. This impaired responsiveness reduces insulin-stimulated glucose uptake in peripheral tissues and weakens the suppression of hepatic glucose production, thereby disrupting systemic glucose homeostasis. IR is a major pathophysiological feature of type 2 diabetes mellitus (T2DM), metabolic dysfunction-associated steatotic liver disease (MASLD), and cardiovascular disease (CVD) (David, Jacqueline & Morris, 2021). Studies show that IR affects 20–40% of adults around the world. Its prevalence continues to increase in parallel with the rising prevalence of obesity and physical inactivity (Valeska et al., 2018). At the cellular level, IR is characterized by impaired insulin receptor signaling and reduced activation of downstream pathways involved in glucose uptake and metabolic regulation. IR is also associated with ectopic lipid accumulation, chronic low-grade inflammation, and impaired cellular energy metabolism (Domenico et al., 2019). These abnormalities further disrupt glucose and lipid homeostasis. IR commonly precedes the clinical onset of T2DM and increases the risk of metabolic and cardiovascular complications (Magdalene, 2019).

Mitochondria are central to cellular energy metabolism through OXPHOS and also contribute to the regulation of intracellular reactive oxygen species (ROS) production and redox homeostasis. Since the early 2000s, mitochondrial dysfunction has been increasingly recognized as an important contributor to IR. Early studies showed that reduced mitochondrial oxidative capacity was associated with impaired metabolic flexibility in skeletal muscle. Because skeletal muscle is the principal site of insulin-stimulated glucose disposal, impaired mitochondrial oxidative capacity in this tissue has profound metabolic consequences (Domenico et al., 2019). Subsequent studies linked IR to impaired electron transport chain (ETC) activity, excessive mitochondrial ROS production, and dysregulated mitochondrial dynamics, including altered fission and fusion (Keenan, Watt & Montgomery, 2020). Beyond these mitochondrial abnormalities, excessive mitochondrial ROS can also promote endoplasmic reticulum (ER) stress, thereby further impairing insulin signaling and responsiveness (Xu et al., 2026). Recent findings further suggest that compromised mitochondrial quality control, including impaired mitophagy and biogenesis, contributes to defective insulin signaling (Takano, Ogawa & Hayakawa, 2023). Taken together, these insights position mitochondrial dysfunction as a central factor in IR pathogenesis and a promising therapeutic target for metabolic diseases.

Although most body iron is incorporated into hemoglobin in circulating erythrocytes and a smaller proportion is stored mainly in ferritin and hemosiderin, stored iron can be mobilized to support cellular iron-dependent processes when required (Abbaspour, Hurrell & Kelishadi, 2014; Ru et al., 2024). Mitochondria use part of this bioavailable iron pool for heme and iron-sulfur (Fe-S) cluster biosynthesis, thereby supporting ETC activity, oxidative phosphorylation (OXPHOS), and redox-dependent metabolic regulation (Dietz, Fox & Khalimonchuk, 2021). Disruption of mitochondrial iron homeostasis can result in either iron overload or iron deficiency, both of which impair mitochondrial energy metabolism through distinct mechanisms. Excess mitochondrial iron promotes reactive oxygen species generation through Fenton chemistry, leading to oxidative damage of mitochondrial proteins, lipids, and mitochondrial DNA. These events compromise mitochondrial bioenergetics, disrupt insulin signaling, and ultimately contribute to the development of IR (Tam et al., 2024). Conversely, mitochondrial iron deficiency restricts Fe-S cluster biosynthesis, thereby compromising ETC activity, reducing ATP production, and impairing energy metabolism in insulin-responsive tissues (Eddie, Hye & Gary, 2023).

Recent studies indicate that dysregulated mitochondrial iron homeostasis contributes to the development of IR and T2DM. In models where a high-fat diet (HFD) causes IR, excessive iron in skeletal muscle exacerbates IR. Mechanistically, excessive mitochondrial iron accumulation promotes mitochondrial reactive oxygen species production, enhances mitochondrial fission, and ultimately impairs insulin signaling (Tam et al., 2024). Moreover, alterations in iron homeostasis may precede the clinical onset of metabolic disease, suggesting that iron dysregulation may participate in the initiation rather than merely the progression of IR (Varghese et al., 2020). A comprehensive understanding of the mechanisms linking mitochondrial iron homeostasis to IR will facilitate the identification of novel therapeutic targets and advance precision interventions for metabolic diseases.

## Survey Methodology

This review is intended for researchers, clinicians, and healthcare professionals in endocrinology and metabolism, as well as investigators in mitochondrial biology and iron homeostasis. It is particularly relevant for readers interested in the mechanistic links between mitochondrial iron dysregulation and IR, and in identifying potential translational targets for T2DM and related metabolic disorders.

A comprehensive literature search was conducted using the PubMed and Web of Science databases, covering publications through July 2026. The search strategy incorporated combinations of free-text keywords and Medical Subject Headings (MeSH) terms where applicable to maximize the retrieval of relevant literature. The representative search categories and keywords used in this review are summarized in Table 1.

The inclusion criteria were as follows: (1) predominantly peer-reviewed articles published in English between 2020 and 2026, with earlier landmark studies included when considered highly relevant; and (2) studies directly related to mitochondrial iron homeostasis, insulin resistance, mitochondrial function, or associated metabolic disorders. The exclusion criteria included case reports, conference abstracts, editorials, commentaries, and studies not directly relevant to the topic. The detailed inclusion and exclusion criteria are summarized in Table 2.

**Table 1:** Literature search strategy used in this review.

Concept	Keywords and MeSH terms
Mitochondrial iron homeostasis	Mitochondrial iron; mitochondrial iron homeostasis; mitochondrial iron metabolism; mitochondrial iron transport; mitoferrin (SLC25A37/SLC25A28); DMT1 (SLC11A2); transferrin receptor (TfR1/TfR2); STEAP3; mitochondrial ferritin (FTMT); ferritinophagy (NCOA4); ferroportin (FPN1/SLC40A1); hepcidin; IRP1; ABCB7; ABCB10; frataxin (FXN); ISCU; Fe-S cluster; heme biosynthesis; FECH; VDAC1; MCU; MitoNEET (CISD1/CISD2); labile iron pool
Insulin resistance	Insulin resistance (IR); insulin signaling; insulin receptor substrate (IRS); IRS-1; Akt; Akt phosphorylation
Metabolic disorders	Type 2 diabetes mellitus (T2DM); prediabetes; obesity; metabolic syndrome; nonalcoholic fatty liver disease (NAFLD)
Mitochondrial function	Electron transport chain (ETC); oxidative phosphorylation (OXPHOS); mitochondrial dysfunction
Oxidative stress	Reactive oxygen species (ROS); oxidative stress; lipid peroxidation; 4-HNE; Nrf2; HO-1
Mitochondrial quality control	Mitochondrial dynamics; DRP1; MFN2; OPA1; mitophagy
ER stress and calcium signaling	ER stress; mitochondria-associated membranes (MAMs); Ca^2^^+^ homeostasis
Inflammation	Inflammation; NF-κB; JNK
Ferroptosis	Ferroptosis; GPX4; iron-dependent cell death
Therapeutic targets	Iron chelator; iron chelation; deferoxamine; deferiprone; deferasirox; antioxidant; resveratrol

**Notes.**

MeSH Medical Subject Headings IR insulin resistance T2DM type 2 diabetes mellitus NAFLD nonalcoholic fatty liver disease ETC electron transport chain OXPHOS oxidative phosphorylation ROS reactive oxygen species MAMs mitochondria-associated membranes Fe-S cluster iron-sulfur cluster

**Table 2:** Inclusion and exclusion criteria for literature selection.

Criteria	Inclusion	Exclusion
Study design	Experimental studies (cell, animal, and human studies) and relevant review articles	Case reports, conference abstracts, editorials, commentaries, and opinion articles
Relevance to research topic	Studies focusing on mitochondrial iron homeostasis and its role in insulin resistance and related metabolic disorders	Studies not related to mitochondrial iron regulation, insulin resistance, or metabolic dysfunction.
Scientific rigor	Peer-reviewed journal articles indexed in major bibliographic databases (PubMed, Web of Science).	Non-peer-reviewed publications
Sample size and statistical methods	Studies with adequate sample size and clear reporting of methods and statistical analyses.	Insufficient sample size, unclear methods/statistics, or incomplete outcome reporting.
Language	Articles published in English.	Articles not in English or with insufficient translation accuracy.
Publication date	Articles published between 2020 and 2026, with earlier studies included if highly pertinent.	Articles published before 2020 unless highly pertinent to the review topic.

After retrieval, titles and abstracts were initially screened for relevance to the research topic. Eligible studies subsequently underwent full-text evaluation, and only studies directly addressing the role of mitochondrial iron homeostasis in IR and related metabolic dysfunction were included in this review.

## Metabolic regulation of mitochondrial iron

Mitochondria serve as the primary intracellular site of iron utilization, incorporating iron into heme and Fe-S clusters that are indispensable for OXPHOS, electron transport, and cellular respiration (Ali et al., 2022). Because both iron deficiency and iron excess impair mitochondrial function through distinct mechanisms, mitochondrial iron homeostasis must be precisely regulated through coordinated iron uptake, utilization, storage, export, and inter-organelle trafficking. Mitoferrins constitute the principal iron transporters across the inner mitochondrial membrane, while additional context-dependent pathways also contribute to mitochondrial iron acquisition. Experimental studies indicate that the mitochondrial calcium uniporter (MCU) can contribute to mitochondrial Fe^2^^+^ uptake under oxidative or iron-overload conditions (Fefelova et al., 2023; Hu et al., 2023; Shui et al., 2021). In addition, endosome–mitochondria interactions can mediate Divalent Metal Transporter 1 (DMT1) dependent mitochondrial iron translocation (Barra et al., 2024), whereas direct lysosome-to-mitochondria Fe^2^^+^ transfer has been demonstrated at mitochondria–lysosome contact sites (Rizzollo et al., 2025) (Fig. 1).

**Figure 1:**
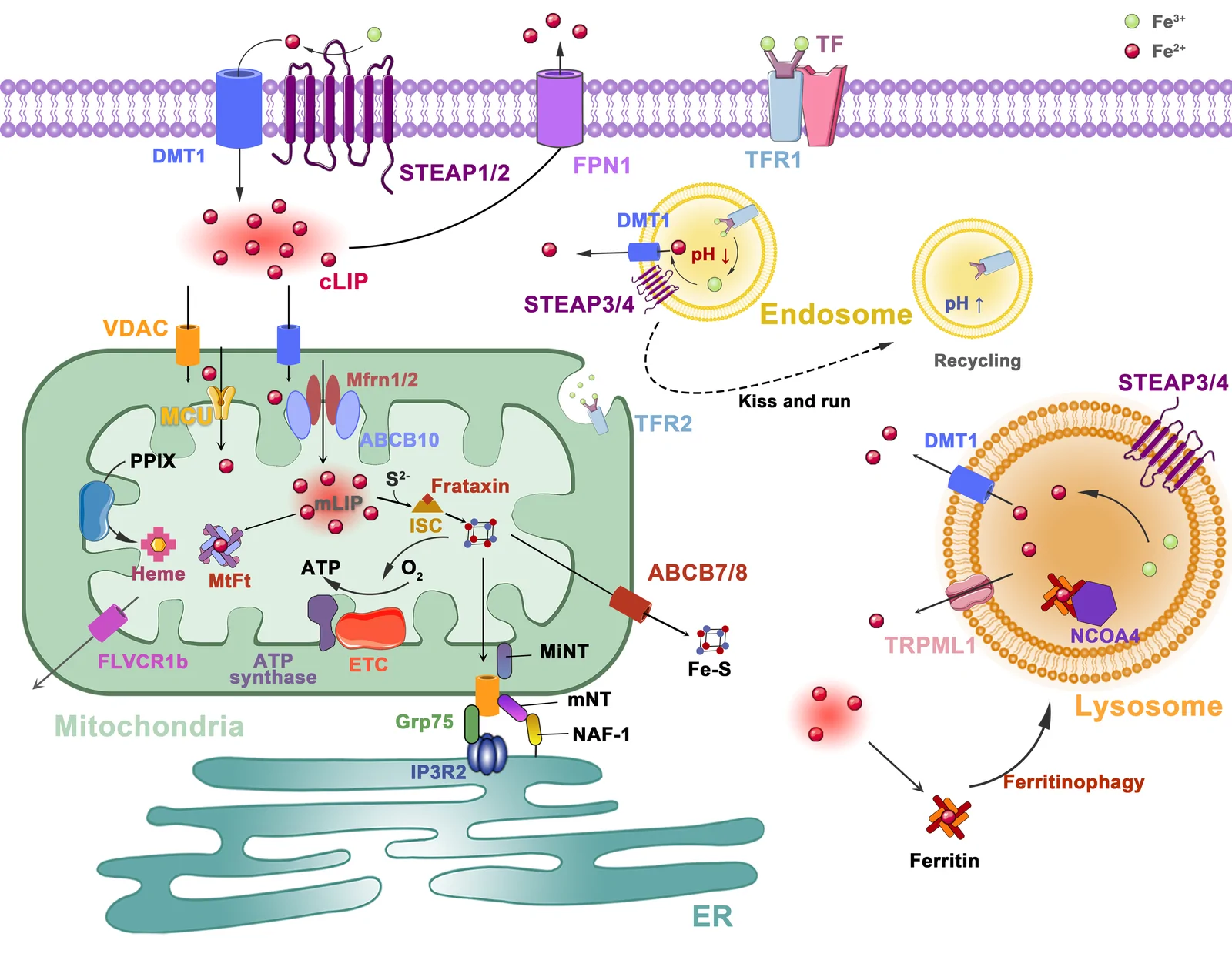
Mitochondrial iron transport and utilization. Transferrin-bound ferric iron (Tf-Fe^3+^) is internalized predominantly through transferrin receptor-mediated endocytosis and reduced to ferrous iron (Fe^2+^) by STEAP3/4 within acidified endosomes. Fe^2+^ is subsequently released into the cytosolic labile iron pool (cLIP) through DMT1. In parallel, plasma membrane STEAP-DMT1 pathways contribute to non-transferrin-bound iron uptake. NCOA4-mediated ferritinophagy mobilizes ferritin-derived iron for redistribution within the intracellular labile iron pool. Transient DMT1-dependent endosome–mitochondria interactions can provide an additional context-dependent route for mitochondrial iron translocation, whereas Mfrn1/2 constitute the principal iron transporters across the IMM, with Mfrn1 functionally stabilized by ABCB10. MCU can contribute to mitochondrial Fe^2+^ uptake under oxidative or iron-overload conditions, although the precise transport mechanism remains incompletely resolved. VDAC1 participates in the IP3R-GRP75-VDAC1-MCU Ca^2+^-signaling axis and in MiNT-VDAC1-mitoNEET-mediated [2Fe-2S] cluster trafficking, rather than being established as a canonical mitochondrial Fe^2+^ importer. Within mitochondria, excess iron is buffered by FtMt and utilized for heme and Fe-S cluster biosynthesis. Heme is synthesized through insertion of Fe^2+^ into PPIX and exported from mitochondria by FLVCR1b. Fe-S cluster biogenesis involves the ISC machinery, including FXN and ISCU. ABCB7 participates in the export of Fe-S-related species required for cytosolic Fe-S protein maturation, whereas ABCB8 contributes to mitochondrial iron export. At the plasma membrane, FPN1 mediates cellular iron efflux and thereby indirectly contributes to mitochondrial iron balance. Abbreviations: ABCB7/8, ATP-binding cassette subfamily B members 7/8; ABCB10, ATP-binding cassette subfamily B member 10; cLIP, cytosolic labile iron pool; DMT1, divalent metal transporter 1; ER, endoplasmic reticulum; Fe-S, iron-sulfur; FLVCR1b, feline leukemia virus subgroup C receptor-related protein 1b; FPN1, ferroportin; FtMt, mitochondrial ferritin; FXN, frataxin; Grp75, glucose-regulated protein 75; IMM, inner mitochondrial membrane; IP3R2, inositol 1,4,5-trisphosphate receptor type 2; ISC, iron-sulfur cluster; ISCU, iron-sulfur cluster assembly enzyme; MAM, mitochondria-associated membrane; MCU, mitochondrial calcium uniporter; Mfrn1/2, mitoferrin 1/2; mNT, MitoNEET; NAF-1, nutrient-deprivation autophagy factor-1; OMM, outer mitochondrial membrane; PPIX, protoporphyrin IX; STEAP, six-transmembrane epithelial antigen of prostate; TfR, transferrin receptor; TRPML1, transient receptor potential mucolipin 1; VDAC, voltage-dependent anion channel.

### Mitochondrial iron uptake

Following cellular entry, iron is distributed to distinct intracellular compartments according to metabolic demand. Mitochondria constitute the principal destination for iron utilization, where imported iron is incorporated into heme and Fe-S clusters required for multiple mitochondrial metabolic processes. Structurally, mitochondria possess a double-membrane architecture consisting of an outer mitochondrial membrane (OMM), an intermembrane space (IMS), an inner mitochondrial membrane (IMM), and the matrix, each contributing to coordinated iron trafficking and utilization. The OMM, owing to the presence of voltage-dependent anion channels (VDACs), is highly permeable to small uncharged molecules and ions, allowing the passage of metabolites such as ATP, cations, and pyruvate. In contrast, the IMM acts as a selective barrier, restricting ion diffusion and relying on specialized hydrophobic mitochondrial carrier proteins to mediate transport across it (Werner, 2015).

#### Classical mitochondrial iron uptake pathways

Mitoferrins (MFRN1/SLC25A37 and MFRN2/SLC25A28) are the principal mitochondrial iron importers responsible for transporting ferrous iron across the IMM, thereby supplying iron for heme synthesis and Fe-S cluster biogenesis. The identification of mitoferrins originated from studies investigating the molecular mechanisms of erythropoiesis. In Shaw et al. (2006) identified MFRN1 as an essential mitochondrial iron transporter required for erythroid iron assimilation and heme biosynthesis, establishing mitochondrial iron import as a critical step in erythroid development. Subsequent studies further expanded the mitoferrin family and demonstrated that mitochondrial iron transport is required not only for erythropoiesis but also for maintaining mitochondrial metabolism in diverse tissues under physiological and pathological conditions (Ali et al., 2022; Geyan et al., 2023).

Beyond its canonical role in erythropoiesis, emerging evidence indicates that MFRN1 also contributes to mitochondrial metabolic remodeling under conditions of metabolic stress. In a HFD-fed obesity-resistant mouse model, MFRN1 was markedly upregulated in gastrocnemius muscle, accompanied by increased citrate synthase and pyruvate dehydrogenase kinase 4 expression, oxidative type IIa fiber remodeling, and a lower fat-to-lean mass ratio (Milhem et al., 2024). These findings suggest that MFRN1 upregulation reflects an adaptive enhancement of mitochondrial iron utilization that supports oxidative remodeling in skeletal muscle during lipid overload. Increased mitochondrial iron availability facilitates the biosynthesis of heme and Fe-S clusters, thereby supporting respiratory-chain complexes and Fe-S-dependent enzymes, including aconitase in the tricarboxylic acid cycle (Ali et al., 2022; Austin et al., 2021). Consequently, elevated MFRN1 expression may promote lipid-derived substrate oxidation rather than simply reflecting enhanced electron transport. Because excessive mitochondrial iron can increase reactive oxygen species generation, the concurrent induction of UCP2 and OPA1 in obesity-resistant muscle may represent a compensatory mechanism that preserves mitochondrial redox homeostasis and mitochondrial dynamics during oxidative adaptation (Ali et al., 2022; Geyan et al., 2023). MFRN1 also physically interacts with the ATP-binding cassette transporter ABCB10, which stabilizes MFRN1 and prolongs its functional activity within the mitochondrial membrane, thereby supporting efficient mitochondrial iron import during periods of high heme demand (Chen et al., 2009).

MFRN2 is expressed in multiple non-erythroid tissues and contributes to the maintenance of mitochondrial iron homeostasis, oxidative phosphorylation, and metabolic flexibility. By transporting ferrous iron into the mitochondrial matrix, MFRN2 supplies iron required for Fe-S cluster biogenesis and mitochondrial respiration, processes that are particularly important in metabolically active tissues such as skeletal muscle, liver, and neurons (Ali et al., 2022; Shao et al., 2024). Recent studies suggest that MFRN2 expression is dynamically regulated during mitochondrial stress. For example, elevated MFRN2 expression has been observed in bleomycin-induced pulmonary fibrosis, in which increased mitochondrial iron accumulation was associated with mitochondrial dysfunction and alveolar epithelial cell injury, suggesting a compensatory response aimed at maintaining mitochondrial iron metabolism (Shao et al., 2024). In addition, dysregulation of MFRN2 has been implicated in metabolic disorders. Overexpression of SLC25A28 (MFRN2) promotes adipogenesis through suppression of adipose triglyceride lipase (ATGL), resulting in increased lipid accumulation and impaired metabolic homeostasis, highlighting a potential role for MFRN2-mediated mitochondrial iron metabolism in obesity-related metabolic diseases (Guan et al., 2024).

DMT1 (also known as SLC11A2) plays an important role in intracellular Fe^2^^+^ trafficking. Its proton-coupled transport activity in acidified endosomal compartments facilitates the release of Fe^2^^+^ into the cytosol, thereby contributing to the intracellular iron pool available for subsequent mitochondrial utilization (Ali et al., 2022; Dietz, Fox & Khalimonchuk, 2021). Beyond this canonical route, transient endosome–mitochondria “kiss-and-run” interactions can support mitochondrial iron translocation. Barra et al. (2024) demonstrated that DMT1 sustains mitochondrial iron translocation through early endosome–mitochondria interactions in MDA-MB-231 cells, whereas this effect was less evident in T47D cells, indicating that the contribution of this pathway may be cell-context dependent. DMT1 has also been detected on the OMM, further supporting its potential involvement in inter-organelle iron trafficking (Wolff et al., 2014). Functionally, DMT1 silencing in MDA-MB-231 cells increased the cytosolic labile iron pool, altered mitochondrial bioenergetics, and promoted PINK1/Parkin-dependent mitophagy, whereas DMT1 re-expression restored mitochondrial iron translocation and bioenergetic function (Barra et al., 2024). In HFD-fed mice and palmitate-treated MIN6 cells, lipotoxicity was associated with increased DMT1 expression together with altered iron metabolism, increased ROS production, and β-cell dedifferentiation (Li et al., 2026).

#### Membrane channel-mediated iron uptake

Voltage-dependent anion channel 1 (VDAC1) is a major OMM porin that connects the mitochondrial IMS with the cytosol and regulates the exchange of ions and metabolites across the OMM. In addition to its general role in mitochondrial metabolite transport, VDAC1 has been implicated in iron-sulfur cluster trafficking. Karmi et al. (2022) identified a VDAC1-mediated NEET protein pathway involving mitochondrial inner NEET protein (MiNT/CISD3), VDAC1, and the OMM-localized mitoNEET (mNT/CISD1). In this pathway, MiNT transfers its labile [2Fe-2S] cluster from the mitochondrial interior through a VDAC1-dependent interaction to mNT facing the cytosol, thereby providing a route for Fe-S cluster transfer from mitochondria toward cytosolic acceptor proteins.

Combined experimental and computational analyses support the formation of the MiNT-VDAC1-mNT protein chain and its role in [2Fe-2S] cluster transfer. Altering MiNT expression also affects mitochondrial ROS levels, mitochondrial morphology, and mitochondrial function, further linking this pathway to mitochondrial homeostasis (Karmi et al., 2022). Separately, VDAC1 deficiency has been shown to alter mitochondrial oxygen consumption and electron transport chain organization, particularly Complex I activity (Magrì et al., 2023). Thus, current evidence supports a role for VDAC1 in Fe-S cluster trafficking and mitochondrial metabolic regulation, whereas direct evidence establishing VDAC1 as a canonical pathway for mitochondrial Fe^2^^+^ import remains limited.

Mitochondria-associated membranes (MAMs) are specialized regions of contact between mitochondria and the ER that participate in Ca^2^^+^ signaling, lipid metabolism, and mitochondrial function (Ji et al., 2022). At these contact sites, inositol 1,4,5-trisphosphate receptors (IP3Rs) on the ER are functionally coupled to VDAC1 on the OMM through glucose-regulated protein 75 (GRP75), facilitating Ca^2^^+^ transfer from the ER toward mitochondria. Ca^2^^+^ subsequently enters the mitochondrial matrix through the MCU complex located in the IMM (Yoo, 2022; Yuan et al., 2022). In this context, Ca^2^^+^ acts as an important second messenger linking ER signaling with mitochondrial metabolic regulation.

Beyond its established function in mitochondrial Ca^2^^+^ uptake, accumulating experimental evidence indicates that MCU can also contribute to mitochondrial Fe^2^^+^ uptake under specific pathological conditions. Shui et al. (2021) observed a reduction in the cytosolic labile iron pool accompanied by an increase in mitochondrial Fe^2^^+^ following photodynamic therapy, whereas pharmacological inhibition of MCU attenuated this iron redistribution. Subsequent genetic studies provided further evidence: cardiac-specific MCU deficiency reduced mitochondrial iron accumulation during iron overload, whereas global or hepatocyte-specific MCU deficiency suppressed mitochondrial Fe^2^^+^ uptake following acetaminophen-induced lysosomal iron release (Fefelova et al., 2023; Hu et al., 2023). Collectively, these findings support an MCU-dependent contribution to mitochondrial Fe^2^^+^ uptake under oxidative, iron-overload, or toxic stress conditions. However, the precise mechanism remains incompletely resolved, and current evidence does not establish that Fe^2^^+^ necessarily permeates directly through the MCU pore.

Additionally, lysosomes are key organelles for intracellular iron storage, recycling, and redistribution. Recent evidence provides direct support for contact-dependent lysosome-to-mitochondria Fe^2^^+^ transfer. Rizzollo et al. (2025) demonstrated that 3-hydroxybutyrate dehydrogenase 2 (BDH2) localizes at mitochondria–lysosome contacts (MLCs), where it generates the mammalian siderophore 2,5-dihydroxybenzoic acid (2,5-DHBA), which facilitates Fe^2^^+^ transfer from lysosomes to mitochondria. Loss of BDH2 resulted in lysosomal iron sequestration, altered MLC dynamics, and reduced mitochondrial iron availability, providing direct mechanistic evidence for inter-organelle Fe^2^^+^ redistribution. Six-transmembrane epithelial antigen of the prostate 3 (STEAP3) functions through a distinct endosomal iron-processing pathway. STEAP3 is an endosomal ferrireductase that converts Fe^3^^+^ to Fe^2^^+^ following transferrin receptor-mediated endocytosis, thereby facilitating DMT1-dependent release of Fe^2^^+^ from acidified endosomal compartments (Ohgami et al., 2005; Ohgami et al., 2006).

#### Indirect mitochondrial iron uptake pathways

Iron homeostasis is also maintained through transferrin (Tf)-mediated iron uptake. Iron-bound transferrin (holo-Tf) interacts with transferrin receptors on the cell surface, of which transferrin receptor 1 (TfR1) is widely expressed and serves as the major high-affinity receptor for cellular transferrin-bound iron uptake. Transferrin receptor 2 (TfR2) has a lower affinity for holo-Tf and is expressed predominantly in hepatocytes and erythroid precursor cells, where it has important roles in systemic iron sensing and erythroid iron regulation (Mleczko-Sanecka & Silvestri, 2021). Following TfR1-mediated endocytosis, endosomal acidification promotes iron release from transferrin, Fe^3^^+^ is reduced to Fe^2^^+^ by ferrireductases such as STEAP3, and DMT1 subsequently transports Fe^2^^+^ out of the endosomal compartment.

In erythroid progenitors, TfR2 can additionally participate in a specialized pathway of iron delivery involving lysosomal trafficking. Khalil et al. (2017) demonstrated that TfR2 redirects transferrin toward lysosomal compartments that interact closely with mitochondria, providing an alternative route for delivering transferrin-derived iron toward mitochondrial heme synthesis. TfR2 depletion reduced heme production and altered mitochondrial morphology, supporting a functional role for this pathway in erythroid iron utilization. Thus, TfR2-dependent lysosomal trafficking appears to represent a specialized, cell-context-dependent mechanism that complements the canonical TfR1-endosome-DMT1 pathway. Once iron becomes available for mitochondrial utilization, transport across the IMM is primarily mediated by mitoferrins, which supply iron for Fe-S cluster and heme biosynthesis (Ali et al., 2022; Geyan et al., 2023).

In addition to the aforementioned iron transport pathways, ferritinophagy provides a mechanism for mobilizing stored cellular iron. Ferritinophagy is a selective autophagic process in which nuclear receptor coactivator 4 (NCOA4) functions as a cargo receptor that delivers ferritin to lysosomes for degradation, thereby releasing ferritin-bound iron into the intracellular labile iron pool (Kuno et al., 2022; Li et al., 2024b). This process is regulated by cellular iron availability and contributes to the redistribution of stored iron toward iron-dependent metabolic processes. In pancreatic cancer cells, NCOA4-mediated ferritinophagy maintains iron bioavailability required for iron-sulfur cluster proteins and mitochondrial metabolism (Santana-Codina et al., 2022). NCOA4 deficiency also disrupts cellular iron homeostasis in hippocampal neuronal HT22 cells, further demonstrating the importance of ferritinophagy in maintaining intracellular iron availability (Bengson et al., 2023).

Ferritinophagy may also functionally intersect with mitochondrial quality-control pathways, although the underlying relationship remains incompletely defined. Mitophagy is commonly regulated through PTEN-induced kinase 1 (PINK1) and Parkin-dependent signaling, while PINK1 deficiency has been shown to perturb the labile iron pool and mitochondrial metab olic homeostasis in colorectal cancer cells (Chen et al., 2023). These findings suggest a functional connection between cellular iron availability and mitochondrial quality control, but do not establish that mitochondrial damage necessarily triggers ferritinophagy to supply iron for newly synthesized mitochondria.

### Mitochondrial iron utilization

Mitochondrial iron plays a pivotal role in cellular metabolism, primarily through its incorporation into Fe-S clusters and heme. These iron-containing cofactors are essential for OXPHOS, electron transport, metabolic signaling, and enzymatic catalysis. The mitochondrion serves as the central hub for Fe-S cluster and heme biosynthesis, ensuring that iron is efficiently utilized while preventing its toxic accumulation. Emerging evidence links Fe-S cluster dysfunction to defective iron-sensing mechanisms and impaired metabolic flexibility, while altered heme biosynthesis is increasingly associated with obesity and vascular diseases (Duerre et al., 2025).

#### Iron-sulfur clusters

Fe-S clusters are essential mitochondrial cofactors that support electron transfer, enzymatic catalysis, redox sensing, and metabolic regulation. Mitochondrial Fe-S cluster synthesis begins with de novo cluster assembly on the iron–sulfur cluster scaffold protein (ISCU). This process is initiated by the cysteine desulfurase NFS1, which mobilizes sulfur from cysteine through its interaction with ISD11 and acyl carrier protein (ACP). Frataxin (FXN) functions as a key regulatory component of the core assembly machinery by facilitating sulfur transfer and promoting efficient cluster formation on ISCU, whereas ferredoxin 2 (FDX2), together with ferredoxin reductase (FDXR), provides the reducing equivalents required for persulfide reduction and [2Fe–2S] cluster assembly. Therefore, mitochondrial Fe–S cluster biogenesis is not merely a passive consequence of iron availability, but a highly coordinated process that requires scaffold proteins, sulfur-donor enzymes, electron-transfer proteins, and regulatory chaperone components (Gervason et al., 2019; Maio, Jain & Rouault, 2020; Perfitt & Martelli, 2022).

After nascent clusters are assembled on ISCU, they must be released and delivered to downstream recipient proteins through dedicated chaperone and transfer systems. The HSPA9-HSC20 chaperone/co-chaperone system facilitates cluster release from ISCU, while glutaredoxin 5 (GLRX5) acts as an early Fe-S cluster transfer factor. Additional maturation proteins, including NFU1 iron–sulfur cluster scaffold homolog, BOLA3, ISCA1, and ISCA2, participate in the maturation and delivery of Fe-S clusters, particularly [4Fe-4S] clusters, to specific mitochondrial client proteins. These client proteins include respiratory chain complexes I, II, and III, as well as tricarboxylic acid cycle enzymes such as mitochondrial aconitase. Thus, mitochondrial iron delivery for Fe-S cluster biogenesis should be understood as a chaperone-guided allocation process that determines whether imported iron is productively incorporated into functional cofactors or remains in the mitochondrial labile iron pool (Maio, Jain & Rouault, 2020; Melber et al., 2016; Perfitt & Martelli, 2022). Recent studies further highlight the interplay between Fe-S cluster biogenesis and mitochondrial energy metabolism. One-carbon metabolism, particularly S-adenosylmethionine (SAM)-dependent methylation, has been shown to influence Fe-S cluster stability and complex I assembly, thereby linking mitochondrial cofactor biogenesis to respiratory competence (Schober et al., 2021).

Fe-S cluster metabolism also coordinates mitochondrial and extra-mitochondrial iron-dependent processes. Cytosolic and nuclear Fe-S protein maturation depends on the mitochondrial iron–sulfur cluster (ISC) machinery and the cytosolic iron-sulfur protein assembly (CIA) pathway. In this process, the mitochondrial ABC transporter ABCB7 is thought to participate in the export of a sulfur-containing or Fe-S-related intermediate required for cytosolic and nuclear Fe-S protein biogenesis, rather than simply exporting complete Fe-S clusters. Disruption of ABCB7 function has been associated with impaired cytosolic Fe-S protein maturation, altered cellular iron regulation, and mitochondrial iron accumulation, as observed in X-linked sideroblastic anemia with ataxia (Andrew et al., 2024; Lill et al., 2015). In parallel, Fe-S cluster status is sensed by iron regulatory protein 1 (IRP1). Under Fe-S-deficient conditions, cytosolic aconitase/IRP1 shifts toward its RNA-binding form, thereby increasing TfR1-dependent iron uptake and limiting iron export or storage programs (Wu et al., 2020). This mechanism provides a direct link between mitochondrial Fe-S cluster biogenesis, cellular iron sensing, and systemic metabolic regulation.

The metabolic relevance of Fe-S cluster biogenesis is particularly important in IR. Impaired Fe-S cluster assembly compromises electron transport chain activity, decreases oxidative phosphorylation efficiency, disrupts aconitase-dependent tricarboxylic acid cycle flux, and promotes mitochondrial reactive oxygen species generation. Conversely, excessive mitochondrial iron that is not efficiently incorporated into Fe-S clusters may expand the mitochondrial labile iron pool and aggravate redox stress. These disturbances can contribute to impaired ATP production, metabolic inflexibility, inflammatory signaling, and defective insulin signal transduction in metabolically active tissues. Consistent with this concept, diabetic peripheral neuropathy models show that downregulated Fe–S cluster biogenesis is associated with impaired mitochondrial ETC function, oxidative stress, and inflammatory injury (Lin et al., 2024). Altered iron homeostasis and mitochondrial dysfunction have also been discussed in the context of obesity and MASLD, although direct evidence linking specific Fe–S cluster assembly defects to these disorders remains limited (Kim, Shin & Yang, 2022). Collectively, Fe–S cluster biogenesis represents a critical functional bridge between mitochondrial iron utilization, respiratory competence, redox homeostasis, and insulin sensitivity.

#### Heme synthesis

Heme biosynthesis is a critical mitochondrial process required for oxygen transport, electron transfer, redox regulation, and mitochondrial respiration. Recent studies have further highlighted the metabolic functions of heme, particularly its role in brown adipose tissue (BAT) bioenergetics, branched-chain amino acid metabolism, and thermogenesis (Duerre et al., 2025). Alterations in heme synthesis or mitochondrial heme handling can therefore affect cellular metabolic.

Heme is synthesized in a multi-step process, beginning in mitochondria with the formation of δ-aminolevulinic acid (ALA) catalyzed by 5-aminolevulinate synthase (ALAS1 and ALAS2). The intermediate coproporphyrinogen III (CoPIII) is then transported back into mitochondria *via* ABCB6, where it is converted into protoporphyrin IX before ferrochelatase (FECH) inserts iron to produce heme (Shetty & Corson, 2020).

Once synthesized, heme must be exported from mitochondria for incorporation into cytosolic and membrane hemoproteins and to prevent excessive intramitochondrial accumulation. FLVCR1b is a mitochondrial isoform of feline leukemia virus subgroup C receptor 1 (FLVCR1) that promotes heme efflux from mitochondria into the cytosol. FLVCR1b overexpression promotes heme synthesis and erythroid differentiation, whereas FLVCR1b silencing causes mitochondrial heme accumulation and impairs erythroid differentiation, providing direct evidence for its role in mitochondrial heme export (Chiabrando et al., 2012).

Mitochondrial heme metabolism is also closely linked to bioenergetic adaptation. In BAT, heme biosynthesis regulates branched-chain amino acid catabolism, mitochondrial respiration, and thermogenic function. Experimental inhibition of heme biosynthesis compromises mitochondrial respiratory capacity and thermogenesis, directly linking mitochondrial heme availability to metabolic adaptation in brown adipocytes (Duerre et al., 2025).

Mitochondrial heme deficiency is now linked to metabolic diseases, including IR, MASLD and vascular dysfunction. A study in endothelial cells demonstrated that heme biosynthesis is essential for nitric oxide (NO) signaling and vascular homeostasis, and its disruption contributes to cardiovascular diseases (Shetty & Corson, 2020). Heme synthesis abnormalities have also been identified in obesity and metabolic syndrome, where impaired mitochondrial heme metabolism leads to oxidative stress, mitochondrial dysfunction, and inflammation (Makinde, Mellick & Feng, 2023). However, direct evidence linking specific defects in mitochondrial heme biosynthesis to IR or MASLD remains comparatively limited. Further studies are therefore required to determine whether mitochondrial heme dysregulation acts as a causal metabolic defect or primarily reflects broader mitochondrial dysfunction in these conditions.

#### Mitochondrial iron storage

Mitochondrial iron storage plays a pivotal role in cellular iron homeostasis, preventing excess free iron from catalyzing harmful ROS production while ensuring sufficient iron availability for Fe-S cluster and heme biosynthesis. The primary mitochondrial iron storage protein, mitochondrial ferritin (FtMt), is uniquely adapted to the mitochondrial environment, acting as a protective reservoir to buffer against fluctuations in iron availability. Unlike cytosolic ferritin, FtMt lacks iron-responsive elements (IREs) in its mRNA, meaning its expression is regulated independently of systemic iron levels and is instead controlled by oxidative stress and hypoxic conditions (Yanatori et al., 2023).

FtMt stores iron in a less reactive mineralized form through its ferroxidase activity, converting Fe^2^^+^ into Fe^3^^+^ for storage within the ferritin mineral core. This process limits the availability of reactive Fe^2^^+^ for Fenton chemistry and thereby reduces iron-dependent ROS generation (Zhang et al., 2020). FtMt expression may also be regulated by antioxidant-response pathways. In particular, activation of nuclear factor erythroid 2-related factor 2 (Nrf2; encoded by *NFE2L2*) has been reported to induce FtMt expression, suggesting that FtMt may participate in an adaptive mitochondrial iron-buffering response under oxidative or ferroptosis-related stress (Ziyi et al., 2026).

Ferritinophagy is a selective autophagic process classically mediated by nuclear receptor coactivator 4 (NCOA4), which delivers cytosolic ferritin to lysosomes for degradation and release of stored iron (Joseph et al., 2014; William et al., 2014). This pathway is best established for cytosolic ferritin. Emerging evidence nevertheless suggests a functional relationship between NCOA4 and FtMt regulation. In primary human macrophages, reduced NCOA4 expression under hypoxia was associated with increased FTMT abundance, and NCOA4 knockdown further increased FtMt abundance (Fuhrmann et al., 2020). In an iron-depletion model, FtMt was also reported to interact directly with NCOA4 and to participate in mitophagy-related responses (Hara et al., 2020). However, these observations do not establish that FtMt undergoes canonical NCOA4-mediated ferritinophagy to the same extent as cytosolic ferritin. FtMt turnover may therefore represent a potential additional mechanism influencing mitochondrial iron redistribution and redox homeostasis, but its molecular basis and physiological relevance remain to be clarified.

Dysregulation of mitochondrial iron storage has been implicated in metabolic disorders, including obesity, type 2 diabetes and MASLD. In obesity, mitochondrial iron overload impairs lipid metabolism, promotes oxidative stress, and exacerbates IR, leading to metabolic dysfunction (Makinde, Mellick & Feng, 2023). In type 2 diabetes-related osteoporosis (T2DOP), FtMt deficiency has been found to promote osteoblastic ferroptosis through excessive mitophagy, indicating a key role of mitochondrial iron sequestration in maintaining bone integrity (Wang et al., 2021). Further, FtMt has been proposed to protect against iron-dependent mitochondrial oxidative injury, suggesting a potential role in metabolic liver disease; however, direct evidence in MASLD remains limited (Yanatori et al., 2023).

### Mitochondrial iron export

SLC40A1 (ferroportin, FPN1) is the only established mammalian cellular iron exporter and mediates iron efflux across the plasma membrane, thereby contributing to systemic and cellular iron homeostasis. FPN1 is tightly regulated by hepcidin, which promotes its internalization and degradation and thereby limits cellular iron release. Disease-causing variants in SLC40A1 can result in ferroportin disease or hemochromatosis type 4 through altered iron-export activity or resistance to hepcidin-mediated regulation (Uguen et al., 2023). Recent evidence further indicates that membrane glycerophospholipids influence the conformational dynamics and iron-export function of FPN1 (Debbiche et al., 2024).

Fe-S clusters synthesized in mitochondria must be exported for cytosolic and nuclear Fe-S protein assembly, a process critical for DNA repair, ribosome biogenesis, and metabolic regulation. ABCB7, a mitochondrial ABC transporter, is responsible for exporting Fe-S cluster precursors in the form of [2Fe-2S] glutathione-bound intermediates (Pearson & Cowan, 2020). Disruption of ABCB7 impairs cytosolic Fe-S protein maturation and is associated with mitochondrial iron accumulation, as observed in X-linked sideroblastic anemia with ataxia (XLSA/A). ABCB7 suppression can also increase mitochondrial ROS and perturb redox-sensitive signaling (Kim, Kim & Kim, 2020). In contrast, ABCB8 has direct experimental support for a role in mitochondrial iron export: ABCB8 downregulation reduces iron export from isolated mitochondria and promotes mitochondrial iron accumulation, whereas ABCB8 overexpression enhances mitochondrial iron export (Ichikawa et al., 2012).

Cellular iron recycling also influences the amount of iron available for mitochondrial utilization or cellular export. NCOA4-mediated ferritinophagy releases ferritin-derived iron into the intracellular labile iron pool (Kuno et al., 2022), whereas plasma membrane FPN1 mediates cellular iron efflux and is regulated by hepcidin (Viveiros et al., 2020). These processes determine cellular iron availability and can thereby indirectly influence mitochondrial iron balance. Excess mitochondrial iron, when not adequately buffered or utilized, can promote ROS generation, lipid peroxidation, and ferroptotic injury (Ru et al., 2024). VDAC1 may additionally influence mitochondrial redox homeostasis through its roles in metabolite exchange and Fe-S cluster trafficking; however, direct evidence that VDAC1-mediated iron efflux prevents ferroptosis in metabolic tissues remains limited (Karmi et al., 2022; Magrì et al., 2023).

## Mitochondrial iron function

Iron is indispensable for cellular metabolism, playing a particularly crucial role in mitochondrial function. Unlike cytosolic iron, which supports diverse enzymatic activities, mitochondrial iron is specifically required for energy production, redox balance, and metabolic signaling. As the primary site of iron utilization, mitochondria incorporate iron into Fe-S clusters and heme, essential cofactors for numerous mitochondrial proteins, including those in ETC and OXPHOS (Dietz, Fox & Khalimonchuk, 2021). Beyond its role in adenosine triphosphate (ATP) synthesis, mitochondrial iron homeostasis is functionally linked to redox control by regulating mitochondrial ROS burden. Excess labile iron can amplify superoxide generation through redox cycling and Fenton chemistry, thereby engaging SOD-mediated antioxidant defenses. In mammalian cells, this superoxide-detoxifying system is mediated by Cu/ZnSODs, including SOD1 and SOD3, and mitochondrial manganese superoxide dismutase (MnSOD/SOD2), rather than iron-dependent SOD enzymes (Lewandowski, Kepinska & Milnerowicz, 2020; Mustapha Umar et al., 2017; Ying et al., 2018). Furthermore, mitochondrial iron influences mitochondrial biogenesis and metabolic flexibility, with studies showing that iron availability regulates peroxisome proliferator-activated receptor gamma coactivator 1-alpha (PGC-1α), nuclear respiratory factors (NRFs), and other iron-responsive signaling networks (Ward & Cloonan, 2019). Collectively, these interconnected functions demonstrate that disturbances in mitochondrial iron homeostasis, whether caused by iron deficiency or iron overload, impair mitochondrial bioenergetics, redox homeostasis, and adaptive metabolism, ultimately leading to mitochondrial dysfunction (Fig. 2).

**Figure 2:**
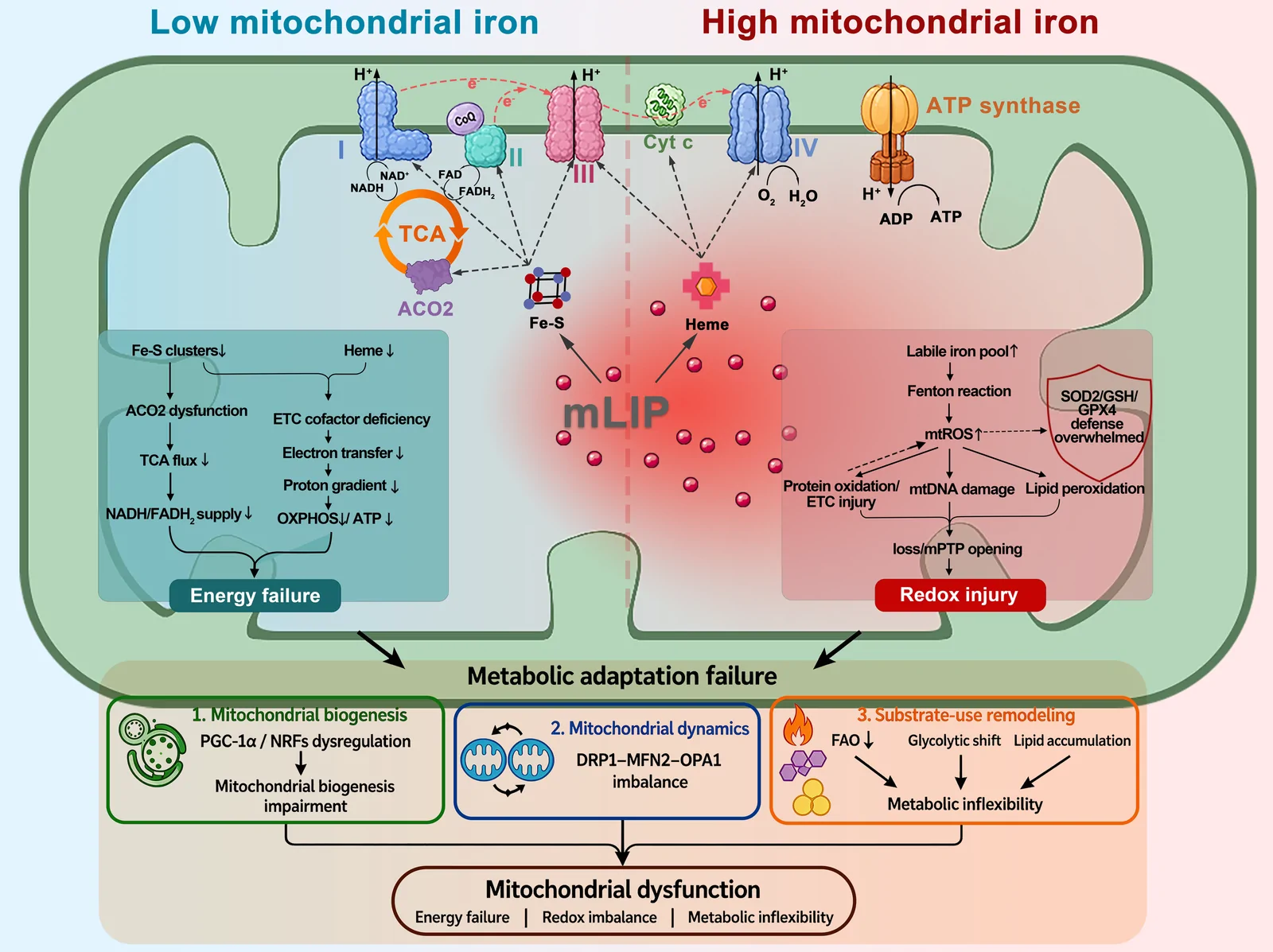
Dual roles of mitochondrial iron homeostasis in mitochondrial function and metabolic adaptation. Mitochondrial iron homeostasis maintains mitochondrial bioenergetics, redox homeostasis, and metabolic adaptation through balanced iron utilization. Mitochondrial iron deficiency impairs Fe–S cluster and heme biosynthesis, resulting in ACO2 dysfunction, reduced TCA cycle flux, impaired ETC electron transfer, decreased OXPHOS activity, ATP depletion, and ultimately energy failure. In contrast, mitochondrial iron overload expands the mLIP, promoting Fenton chemistry and excessive mtROS production. Oxidative stress overwhelms the SOD2/GSH/GPX4 antioxidant system, leading to protein oxidation, mtDNA damage, lipid peroxidation, ETC injury, and mPTP opening, culminating in redox injury. Beyond its roles in bioenergetics and redox regulation, mitochondrial iron also coordinates mitochondrial adaptation by regulating mitochondrial biogenesis, mitochondrial dynamics, and substrate utilization. Consequently, either mitochondrial iron deficiency or overload disrupts mitochondrial adaptation, resulting in metabolic inflexibility and ultimately converging on mitochondrial dysfunction. Abbreviations: ACO2, aconitase 2; ADP, adenosine diphosphate; ATP, adenosine triphosphate; CoQ, coenzyme Q; Cyt c, cytochrome c; DRP1, dynamin-related protein 1; ETC, electron transport chain; FAO, fatty acid oxidation; FADH, reduced flavin adenine dinucleotide; Fe–S, iron–sulfur; GPX4, glutathione peroxidase 4; GSH, glutathione; MFN2, mitofusin 2; mLIP, mitochondrial labile iron pool; mPTP, mitochondrial permeability transition pore; mtDNA, mitochondrial DNA; mtROS, mitochondrial reactive oxygen species; NADH, reduced nicotinamide adenine dinucleotide; NRFs, nuclear respiratory factors; OPA1, optic atrophy protein 1; OXPHOS, oxidative phosphorylation; PGC-1 a, peroxisome proliferator-activated receptor gamma coactivator-1 alpha; SOD2, superoxide dismutase 2; TCA, tricarboxylic acid.

### Mitochondrial iron in OXPHOS

Mitochondrial oxidative phosphorylation (OXPHOS) is the principal mechanism of ATP production in eukaryotic cells and depends on the coordinated activity of the ETC embedded within the inner mitochondrial membrane. Iron is indispensable for OXPHOS because it is incorporated into Fe-S clusters and heme-containing cytochromes, which mediate electron transfer and sustain mitochondrial bioenergetic function (Dietz, Fox & Khalimonchuk, 2021).

Fe-S clusters serve as essential redox cofactors for ETC complexes I, II, and III, mediating electron transfer between catalytic centers and thereby supporting efficient ATP synthesis. Disruption of Fe-S cluster assembly, as observed in iron deficiency or mitochondrial dysfunction, leads to impaired ETC activity and reduced ATP production (Dietz, Fox & Khalimonchuk, 2021; Maio, Jain & Rouault, 2020; Perfitt & Martelli, 2022). Fe-S clusters are also required for mitochondrial aconitase 2 (ACO2), a tricarboxylic acid (TCA)-cycle enzyme that catalyzes the reversible conversion of citrate to isocitrate. Because ACO2 activity depends on an intact [4Fe-4S] cluster, impaired Fe-S cluster biogenesis or oxidative disruption of Fe-S clusters may affect both ETC activity and TCA-cycle flux, linking mitochondrial iron availability to broader mitochondrial metabolic regulation (Padalko, Posnik & Adamczyk, 2024; Perfitt & Martelli, 2022). Heme provides the prosthetic group for cytochromes within Complexes III and IV, thereby enabling electron transport and terminal oxygen reduction during OXPHOS (Kim, Shin & Yang, 2022). Perturbations in mitochondrial iron availability impair heme biosynthesis, compromise ETC efficiency, and promote compensatory metabolic remodeling toward glycolytic ATP production (Li et al., 2019). Dysfunctional Fe-S cluster biogenesis impairs ETC efficiency, leading to reduced ATP production and increased ROS generation. Mitochondrial iron dysregulation can impair OXPHOS by disrupting iron-dependent respiratory components, and impaired mitochondrial respiration may promote compensatory metabolic remodeling toward increased glycolytic dependence. In metabolic stress models, mitochondrial iron overload has also been linked to increased ROS generation, mitochondrial dysfunction, and impaired insulin signaling (Eddie, Hye & Gary, 2023; Li et al., 2019). In addition, cytochrome c (Cyt c) is a mobile iron-containing electron carrier that shuttles electrons between Complex III (cytochrome bc1) and Complex IV (cytochrome c oxidase, CytOx), ensuring efficient ATP production. Post-translational phosphorylation of Cyt c modulates electron-transfer efficiency and apoptotic susceptibility, thereby functionally linking mitochondrial respiration with cell-fate regulation (Kalpage et al., 2020).

Maintenance of mitochondrial iron homeostasis is equally important for preventing both iron deficiency and iron overload, as either condition compromises respiratory function through distinct mechanisms. Excess mitochondrial iron promotes ROS generation through Fenton chemistry, resulting in oxidative injury to ETC complexes, mitochondrial phospholipids, and mitochondrial DNA (Yan et al., 2022). These events facilitate mitochondrial permeability transition pore (MPTP) opening, membrane depolarization, and bioenergetic failure (Fedotcheva, Shimanovsky & Fedotcheva, 2023). In conditions such as iron overload disorders or ferroptosis, excessive mitochondrial iron induces lipid peroxidation, further exacerbating mitochondrial dysfunction (Li et al., 2024a). Emerging evidence suggests that iron availability modulates mitochondrial metabolic plasticity, influencing the balance between OXPHOS and glycolysis. Conversely, iron depletion activates hypoxia-inducible factor (HIF)-dependent metabolic remodeling, suppressing OXPHOS while enhancing glycolytic metabolism to preserve cellular ATP production under conditions of impaired mitochondrial respiration (Li et al., 2019). This metabolic shift is often observed in diseases associated with mitochondrial dysfunction, including type 2 diabetes and neurodegeneration, where altered iron homeostasis contributes to bioenergetic inefficiency and increased reliance on non-mitochondrial ATP production (Ward & Cloonan, 2019).

Collectively, these findings demonstrate that precise mitochondrial iron homeostasis is indispensable for maintaining respiratory efficiency, ATP generation, and metabolic flexibility, whereas either iron deficiency or iron overload disrupts mitochondrial bioenergetics and predisposes cells to metabolic dysfunction.

### Mitochondrial iron in redox homeostasis

Mitochondria are the predominant intracellular source of ROS, with electron leakage occurring primarily at ETC complexes I and III during oxidative phosphorylation. Experimental evidence demonstrates that oxidative degradation of Fe-S clusters compromises mitochondrial respiration, ATP production, and metabolic flexibility (Ježek et al., 2024). Additionally, iron-dependent cytochrome c oxidase (COX), a critical component of the ETC, requires precise iron homeostasis for proper assembly and function. Defects in COX biogenesis disrupt mitochondrial respiration and alter cellular metal homeostasis, further impairing redox balance (Brischigliaro et al., 2022).

Recent studies have identified an iron-glutathione (GSH) autoregulatory mechanism that directly links mitochondrial iron homeostasis with antioxidant defense (Ali et al., 2022; Liu et al., 2023). Under iron-deficient conditions, stabilization of the mitochondrial glutathione transporter SLC25A39 enhances GSH import into mitochondria, thereby strengthening antioxidant capacity. Conversely, iron overload disrupts SLC25A39 regulation, leading to impaired antioxidant defenses and heightened susceptibility to ferroptosis, an iron-dependent form of cell death (Geyan et al., 2023). Mitochondrial antioxidant defenses are supported by MnSOD/SOD2 and glutathione peroxidase 4 (GPX4). SOD2 catalyzes the dismutation of superoxide (O_2_^−^) into hydrogen peroxide (H_2_O_2_) and molecular oxygen, whereas GPX4 is a selenium-dependent enzyme that uses GSH to reduce phospholipid hydroperoxides and suppress ferroptosis (Lewandowski, Kepinska & Milnerowicz, 2020; Ru et al., 2024; Ying et al., 2018). Although neither SOD2 nor GPX4 uses iron as a catalytic cofactor, mitochondrial iron overload can overwhelm these antioxidant systems by increasing ROS production and disrupting GSH-dependent redox homeostasis, thereby promoting lipid peroxidation and ferroptotic injury. Such redox dysregulation has been implicated in obesity, IR, and MASLD (Kim, Shin & Yang, 2022).

### Mitochondrial iron in cellular adaptation and energy sensing

Beyond its roles in OXPHOS and redox regulation, mitochondrial iron also orchestrates mitochondrial adaptation by modulating biogenesis, dynamics, and metabolic flexibility in response to changing energetic demands (Kim, Shin & Yang, 2022).

Mitochondrial biogenesis, the process of generating new mitochondria, is tightly regulated by PGC-1α and NRFs. Iron availability directly modulates PGC-1α -NRFs-mediated transcriptional regulation, thereby coupling mitochondrial iron status with mitochondrial biogenesis and respiratory capacity (Zhou et al., 2024). Conversely, iron supplementation enhances mitochondrial biogenesis, promoting increased maximal oxygen uptake (VO2 max) and ATP synthesis. This suggests that iron plays a crucial role in adapting mitochondrial function to metabolic demands, particularly in tissues with high energy requirements such as skeletal muscle (Fischer et al., 2022).

Mitochondria undergo continuous fission and fusion to adapt to cellular energy demands. Dynamin-related protein 1 (DRP1)-mediated fission and mitofusin (MFN2) and OPA1-mediated fusion are essential for mitochondrial quality control. Iron availability can modulate these processes under iron-deficient conditions. In developing hippocampal neurons, iron deficiency has been shown to impair dendritic mitochondrial motility, decrease mitochondrial size, and alter fusion- and fission-related gene expression, suggesting that insufficient iron availability may disrupt mitochondrial dynamics and morphology through impaired bioenergetic capacity (Thomas et al., 2019). Conversely, mitochondrial iron overload can aggravate ROS generation, mitochondrial dysfunction, and metabolic stress, thereby disturbing mitochondrial quality-control processes and contributing to impaired metabolic homeostasis (Eddie, Hye & Gary, 2023). Together, these findings suggest that mitochondrial iron imbalance is linked to altered mitochondrial morphology and dynamics in a context-dependent manner (Eddie, Hye & Gary, 2023; Thomas et al., 2019).

Mitochondrial iron also dictates metabolic flexibility, the ability of cells to switch between glycolysis and fatty acid oxidation (FAO) based on energy needs. Disruption of iron-regulatory signaling can shift cellular metabolism from OXPHOS toward aerobic glycolysis through hypoxia-inducible factor 1α/2α (HIF1α/HIF2α)-dependent remodeling of Fe-S cluster biogenesis and ETC-related gene expression. Consistently, iron deficiency has been associated with reduced pyruvate dehydrogenase flux, increased lactate production, and induction of glycolytic enzyme-related programs, reflecting a compensatory glycolytic phenotype when mitochondrial oxidative metabolism is compromised. Conversely, mitochondrial iron overload can increase mitochondrial iron burden, impair mitochondrial function, and weaken insulin signaling, thereby limiting metabolic flexibility in insulin-responsive tissues. These findings suggest that mitochondrial iron homeostasis regulates metabolic flexibility through coordinated effects on Fe–S-dependent respiration, HIF-driven glycolytic remodeling, and mitochondrial iron overload-associated IR (Eddie, Hye & Gary, 2023; Jianyu et al., 2023; Li et al., 2019; Yu Jin et al., 2021).

Overall, mitochondrial iron coordinates mitochondrial biogenesis, dynamics, and substrate selection through integrated regulation of PGC-1α-NRFs-mediated transcriptional programs, DRP1-MFN2-OPA1-mediated mitochondrial remodeling, and HIF-dependent metabolic adaptation. Dysregulation of mitochondrial iron homeostasis disrupts these adaptive responses, thereby promoting metabolic inflexibility and disease progression.

## Mitochondrial iron and IR

Mitochondrial iron homeostasis is essential for maintaining metabolic balance, and its disruption has emerged as an important contributor to the development of IR. Beyond serving as a cofactor for mitochondrial metabolism, iron directly regulates oxidative phosphorylation, redox homeostasis, and insulin-responsive bioenergetic adaptation. Consequently, excessive mitochondrial iron promotes oxidative stress, mitochondrial dysfunction, and impaired insulin signaling, thereby contributing to metabolic dysregulation (Tam et al., 2024).

Excess mitochondrial iron impairs ETC efficiency, leading to electron leakage and the overproduction of ROS, particularly at Complex I and Complex III. The resulting ROS accumulation promotes oxidative stress and lipid peroxidation, two major pathological processes driving IR progression (Blagov et al., 2024). The Fenton reaction, which involves Fe^2^^+^ reacting with hydrogen peroxide (H_2_O_2_) to generate hydroxyl radicals (•OH), further exacerbates oxidative damage. Oxidative stress can activate stress-sensitive kinases that promote inhibitory serine phosphorylation of insulin receptor substrate 1 (IRS-1), thereby attenuating PI3K-Akt signaling and insulin-stimulated glucose uptake (Copps & White, 2012). Supporting the pathogenic role of the mitochondrial iron-ROS axis, MitoNEET-mediated regulation of mitochondrial iron reduces ROS burden and preserves insulin signaling under iron-overload conditions (Eddie, Hye & Gary, 2023).

Although a universal mitochondrial iron concentration threshold for ETC impairment has not been established, mitochondrial labile iron is tightly regulated and can expand under iron-loading conditions, thereby increasing the risk of iron-catalyzed oxidative injury (Sobieska et al., 2024). In iron-overload models, excess iron has been associated with reduced ETC complex II/III activity, decreased ATP production, and ROS-dependent mitochondrial fragmentation through activation of the AMPK-MFF-Drp1 pathway (Qingqing et al., 2018). Consistently, in skeletal muscle cells exposed to iron overload, MitoNEET-mediated regulation of mitochondrial iron reduced mitochondrial ROS accumulation, mitochondrial fission, and mitophagy, thereby preserving insulin sensitivity (Eddie, Hye & Gary, 2023). These findings suggest that mitochondrial iron overload contributes to IR-related mitochondrial dysfunction through redox injury and dysregulated mitochondrial dynamics rather than through a fixed concentration-defined threshold (Eddie, Hye & Gary, 2023; Qingqing et al., 2018).

Mitochondrial iron overload disrupts cellular metabolism through multiple interrelated mechanisms, ultimately contributing to IR. Excess mitochondrial iron disrupts Fe-S cluster biogenesis, thereby compromising ETC activity and mitochondrial bioenergetic efficiency. In addition to ETC impairment, Fe-S cluster-dependent TCA-cycle remodeling may also participate in IR-related metabolic dysfunction. ACO2 is particularly relevant in this context because its catalytic activity depends on an intact [4Fe-4S] cluster and is sensitive to impaired Fe-S cluster biogenesis and oxidative cluster disruption. Reduced ACO2 activity may attenuate citrate-isocitrate interconversion, limit TCA-cycle flux, and decrease NADH/FADH_2_ supply for downstream OXPHOS. In insulin-responsive tissues, such bioenergetic restriction may contribute to impaired oxidative substrate utilization and metabolic inflexibility under nutrient excess conditions (Padalko, Posnik & Adamczyk, 2024). Simultaneously, iron accumulation catalyzes lipid peroxidation, generating toxic byproducts such as 4-hydroxynonenal (4-HNE), which impair insulin signaling. Moreover, ferroptosis, an iron-dependent form of cell death, exacerbates IR by damaging pancreatic β-cells and reducing insulin secretion (Ru et al., 2024). Although direct evidence linking ACO2 dysfunction to IR remains limited, current findings support ACO2 as a plausible Fe-S-dependent metabolic node through which mitochondrial iron dysregulation may contribute to IR-associated bioenergetic remodeling.

In addition to impairing mitochondrial bioenergetics, mitochondrial iron overload also disrupts MAMs, which function as essential signaling platforms coordinating lipid metabolism, calcium homeostasis, and insulin signaling (Logan, Henver & Marcelo, 2020). Iron-induced oxidative stress further destabilizes MAM integrity, activates NF-κB- and JNK-dependent inflammatory signaling, and exacerbates IR (Galizzi & Di Carlo, 2022). Collectively, these disruptions establish a vicious cycle in which mitochondrial dysfunction, oxidative stress, and inflammation reinforce one another, driving the progression of IR and metabolic dysregulation.

Evidence from cellular, animal, and clinical studies consistently supports the pathogenic role of mitochondrial iron overload in IR. In skeletal muscle cells, *in vitro* studies using L6 myocytes demonstrated that excess iron leads to mitochondrial iron accumulation and elevated ROS production. This oxidative stress disrupts insulin signaling by reducing insulin-stimulated Akt phosphorylation, ultimately impairing glucose uptake (Tam et al., 2024).

Similarly, in animal models, iron overload in mice was associated with increased mitochondrial iron concentrations, mitochondrial fission, and oxidative stress, all of which contributed to IR. Notably, the overexpression of CDGSH Iron-Sulfur Domain-Containing Protein 1 (MitoNEET, CISD1), a mitochondrial iron regulator, mitigated these effects and improved insulin sensitivity, highlighting a potential therapeutic target for metabolic dysfunction (Eddie, Hye & Gary, 2023).

Human observational studies have associated elevated serum ferritin with higher fasting insulin levels and impaired glucose tolerance, suggesting a relationship between systemic iron status and IR (Sobieska et al., 2024). Together, these complementary findings across experimental systems and human studies strongly support mitochondrial iron overload as a mechanistic driver of IR and metabolic dysfunction.

## Targeted Therapy Based on Mitochondrial Iron

Restoration of mitochondrial iron homeostasis has emerged as a potential therapeutic strategy for IR and related metabolic disorders. By limiting iron-dependent oxidative injury, preserving mitochondrial bioenergetics, and maintaining insulin signaling, interventions targeting iron accumulation or its downstream consequences may improve metabolic homeostasis. Current approaches include iron chelation, mitochondria-targeted antioxidant therapy, and genetic or protein-level modulation of iron-regulatory pathways (Table 3).

**Table 3:** Summary of therapeutic strategies targeting mitochondrial iron homeostasis in IR.

Name	Mechanism	Model	Refs
DFP	Membrane-permeable iron chelator; reduces intracellular iron availability and may alleviate iron-associated oxidative stress	Diabetic rat model	Peña-Montes et al. (2024)
DFO	Classical systemic iron chelator; reduces total body iron, indirectly affects mitochondrial iron	Iron overload and metabolic disease models	Yan et al. (2018)
Novel lipophilic mitochondrial chelators	Selective mitochondrial iron chelation; decreases ROS and ferroptosis, improves insulin signaling	Experimental models (preclinical)	Kontoghiorghes (2022)
SkQ1	Mitochondria-targeted antioxidant; scavenges ROS, prevents iron-induced IR	L6 skeletal muscle cells exposed to iron overload	Tam et al. (2024)
SOD2/Catalase-overexpressing mesenchymal stromal cells	Enhances endogenous mitochondrial antioxidant defenses; lowers ROS, improves insulin sensitivity	Obesity models with stromal cell therapy	Domingues et al. (2019)
Hesperidin	Dietary polyphenol; reduces oxidative stress *via* miR-149 signaling, restores insulin sensitivity	High-glucose-induced cellular IR model	Tian et al. (2021)
HO-1 (adipocyte-specific gene therapy)	Enhances heme metabolism, prevents iron overload; improves mitochondrial and vascular function	Obese mouse models	Singh et al. (2020)
MitoNEET overexpression	Regulates mitochondrial iron homeostasis; lowers ROS and lipid peroxidation, enhances flexibility	L6 skeletal muscle cells and H9c2 cardiomyoblasts exposed to iron overload	Eddie, Hye & Gary (2023) and Tam et al. (2024)
FPN1 modulation	Alters macrophage iron retention and bioenergetics, but did not significantly modify systemic insulin action	Myeloid-specific ferroportin knockout mice.	Winn et al. (2021)

**Notes.**

DFP deferiprone DFO deferoxamine ROS reactive oxygen species ETC electron transport chain FPN1 ferroportin 1 ABCB7 ATP-binding cassette subfamily B member 7 HO-1 heme oxygenase-1 SOD2 superoxide dismutase 2

### Iron chelators: reducing mitochondrial iron overload

Iron chelation therapy provides a potential strategy for alleviating iron-associated oxidative stress and mitochondrial dysfunction, with possible applications beyond conventional iron-overload disorders. Classical iron chelators, including deferiprone (DFP) and deferoxamine (DFO), are clinically used to reduce systemic iron burden, and accumulating experimental evidence suggests that modulation of excessive iron availability may also benefit IR and T2DM-associated metabolic dysfunction (Sobieska et al., 2024; Yan et al., 2018).

Among these agents, DFP is membrane permeable and can access intracellular iron pools more readily than DFO. In a diabetic rat model, DFP preserved mitochondrial bioenergetic function, enhanced antioxidant responses, and reduced mitochondrial oxidative stress (Peña-Montes et al., 2024). These findings support the potential of DFP to attenuate iron-associated mitochondrial injury in diabetes. However, direct evidence that DFP selectively removes mitochondrial iron or consistently improves whole-body insulin sensitivity remains limited. Therefore, its potential metabolic benefits should be interpreted primarily in the context of reducing intracellular iron availability and iron-dependent oxidative stress rather than as an established mitochondria-specific treatment for IR.

DFO is less membrane permeable and predominantly reduces extracellular, circulating, and endosomal iron availability. Nevertheless, reduction of tissue iron burden by DFO may indirectly alleviate oxidative stress, inflammation, and impaired insulin signaling in iron-loaded or obese metabolic states (Sobieska et al., 2024; Yan et al., 2018). Differences in cellular permeability and subcellular distribution between DFP and DFO should therefore be considered when evaluating their metabolic effects.

Beyond conventional chelators, novel mitochondria-targeted lipophilic iron chelators are being developed to selectively sequester mitochondrial iron without disrupting systemic iron metabolism (Kontoghiorghes, 2022). These specialized chelators effectively reduce mitochondrial iron-driven ROS production and ferroptosis, thereby enhancing insulin signaling. However, despite their therapeutic promise, careful dosing remains crucial to prevent systemic iron depletion and anemia, as iron is essential for hemoglobin synthesis and various enzymatic functions. Balancing iron reduction with metabolic homeostasis will be critical for optimizing the clinical utility of iron chelation therapy in metabolic disorders.

### Antioxidant therapy: targeting mitochondrial oxidative stress

Antioxidant therapy represents a strategic approach to counteracting mitochondrial oxidative stress induced by excess iron, thereby preventing cellular damage and preserving insulin sensitivity. Mitochondria-targeted antioxidants, such as SkQ1 and MitoQ, have shown promising effects in mitigating iron-driven metabolic dysfunction (Fiorenza et al., 2024; Tam et al., 2024). SkQ1, a potent mitochondrial ROS scavenger, has been demonstrated to prevent iron overload-induced IR in skeletal muscle cells (Tam et al., 2024). Mitochondria-targeted antioxidants have been proposed as complementary approaches to reduce ROS-mediated metabolic injury. However, their effects are not specific to iron-derived ROS, and direct evidence that they correct mitochondrial iron-driven IR remains limited. Therefore, their therapeutic role should be distinguished from that of interventions directly targeting mitochondrial iron accumulation.

Enhancement of endogenous mitochondrial antioxidant capacity represents a potential strategy for limiting ROS-mediated impairment of insulin signaling. Nevertheless, direct evidence that targeted SOD2 or catalase augmentation corrects mitochondrial iron-driven IR remains limited, and this approach requires further validation in tissue-specific metabolic models (Domingues et al., 2019).

Natural polyphenols may provide an additional, less invasive strategy for reducing oxidative stress and improving insulin signaling. Hesperidin attenuated high-glucose-induced mitochondrial dysfunction, oxidative stress, and abnormalities in IR-related signaling through restoration of miR-149 expression (Tian et al., 2021). Resveratrol has also been associated with improved insulin sensitivity, reduced oxidative stress, and activation of Akt-related signaling in metabolic disease models. However, these compounds have pleiotropic biological effects and do not specifically target mitochondrial iron. Thus, polyphenol-based interventions should be considered adjunctive approaches that interrupt ROS-mediated amplification of IR rather than direct treatments for mitochondrial iron overload.

Collectively, mitochondria-targeted antioxidants, endogenous antioxidant reinforcement, and natural polyphenols may preserve mitochondrial function and insulin signaling by reducing iron-associated oxidative injury. Their efficacy is likely to be greatest when combined with interventions that directly correct excessive iron accumulation or abnormal iron trafficking.

### Genetic and protein-targeted modulation of iron metabolism

Gene therapy is emerging as a promising approach for modulating mitochondrial iron metabolism to counteract IR. One key target is heme oxygenase-1 (HO-1), which plays a crucial role in iron homeostasis and oxidative stress regulation. Adipocyte-specific HO-1 gene therapy improved insulin sensitivity and vascular function in an obese mouse model, effects associated with enhanced antioxidant capacity and reduced metabolic inflammation (Singh et al., 2020). However, whether these benefits are mediated specifically through mitochondrial iron regulation remains to be determined.

Another promising target is MitoNEET, a mitochondrial iron regulator that prevents iron overload-induced metabolic dysfunction. Overexpression of MitoNEET reduces mitochondrial ROS accumulation, limits excessive mitochondrial fission, and decreases lipid peroxidation, ultimately enhancing metabolic flexibility and insulin responsiveness (Tam et al., 2024). Beyond pharmacological approaches, modulation of iron transport proteins represents another potential therapeutic strategy. Manipulation of cellular iron exporters may also influence tissue-specific metabolic phenotypes. However, myeloid-specific deletion of FPN1 impaired macrophage bioenergetics without significantly altering systemic insulin action, indicating that macrophage iron retention alone is insufficient to determine whole-body insulin sensitivity (Winn et al., 2021). Accordingly, the therapeutic relevance of FPN1 modulation in IR remains uncertain and requires tissue-specific validation.

Although genetic and protein-targeted strategies have shown encouraging preclinical potential, clinical translation will require improvements in tissue-specific delivery, gene-editing precision, reversibility, and long-term safety. Particular attention should be paid to avoiding excessive iron depletion or redistribution, because mitochondrial iron is indispensable for OXPHOS, heme synthesis, and Fe-S cluster biogenesis. Carefully controlled modulation, rather than indiscriminate reduction of mitochondrial iron, will be central to the development of effective therapies for IR and related metabolic disorders.

## Conclusions and perspectives

Mitochondrial iron homeostasis plays a fundamental role in cellular metabolism, OXPHOS, redox balance, and metabolic regulation. Dysregulation of mitochondrial iron, whether through iron overload or deficiency, contributes to IR and metabolic disorders, disrupting mitochondrial function, increasing oxidative stress, and impairing insulin signaling. Emerging evidence highlights mitochondrial iron as a potential therapeutic target, with interventions such as iron chelators, antioxidants, and gene therapy showing promise in restoring metabolic homeostasis. Despite significant advancements, several challenges remain. The precise regulatory mechanisms of mitochondrial iron transport and utilization require further investigation, particularly in the context of disease-specific iron dysregulation. Identifying optimal therapeutic windows for iron modulation while avoiding systemic iron depletion is a crucial consideration. Additionally, the development of targeted iron chelators and mitochondria-specific antioxidants holds potential for more precise interventions. Future research should explore the crosstalk between mitochondrial iron and metabolic pathways, particularly its role in lipid metabolism, inflammation, and ferroptosis. Advances in gene-editing technologies may offer new strategies for fine-tuning mitochondrial iron regulation, reducing the risks associated with iron-driven oxidative stress and metabolic dysfunction. By integrating biochemical, genetic, and pharmacological approaches, we can refine therapeutic strategies for IR and metabolic diseases. Addressing these challenges will be key to translating fundamental discoveries into clinical applications, paving the way for novel, mitochondria-targeted treatments that restore metabolic balance.

## Additional Information and Declarations

### Data Availability

The following information was supplied regarding data availability: This is a Literature Review.
